# Th Poison of Tobacco

**Published:** 1889-08

**Authors:** J. M. W. Kitchen


### THE POISON OF TOBACCO.

The great bulk of the evil physical effects due to the moderate use of tobacco are of an intermediate nature and not directly noticeable. The plainly marked results following the use of tobacco in relatively large amounts seem to be due to quick and extreme interference with nutrition, and a diminution of function of all kinds, which may be represented by anything from a slight decrease of appetite and digestive ability up to a complete loss of function of almost any important organ. Alcohol, owing to the usual method of introduction through the stomach, produces directly noticeable structural changes. But with tobacco, the direct evil results are mostly of a functional character, and are more generally diffused, owing to the usual slow manner of introduction into the body. It is easy to see the effects of large amounts of tobacco in the stunted growth of adolescents ; in functional cardiac disorders ; in intellectual sluggishness, loss of memory, and color blindness ; in loss of appetite and other neuroses of motion, and marked blunting of various functions of sensation, and in degeneracy of descendants. The greater evils that are the outcome of a moderate use of tobacco are probably due to prolonged slight interference with nutrition, and consequent general decrease of vitality which renders the individual more susceptible, through indirect influence, to the invasion of disease, and which lessens the capacity for productive effort.—DR. J. M. W. KITCHEN, in *Medical Record.*

